# Selections and Abstracts

**Published:** 1917-04

**Authors:** 


### SELECTIONS AND ABSTRACTS

### LIGHT AS A CAUSE OF DISEASE

By Thos. W. Murrell, M. D·, Richmond, Va.

In this paper I wish to consider some of the many effects of light on the skin, and by analogy suggest them as a part cause of another condition.

Ordinarily sunlight combines two forms of energy; the heat, except when extreme, is purely beneficial and supports life; the chemic actinic part is simply and only destructive, but may be necessary to life as a stimulant. We are all familiar with the phenomenon of sunburn. Here exposure to light induces dermatitis. This will be transitory, for nature prevents its recurrence by transposing a pigment screen which effectually blocks the light and prevents its destructive actionent condition commonly called tanning. We, therefore, have in the body pigment which varies to such an extent that it determines the human family into blonde and brunette, with the far Northerner at one end of the line and the negro at the other. Microscopical sections of the skin of the negro and the white man show but one decided difference and two minor ones. The minor differences are the increase of the sebaceous glands and the decrease of the lanugo hairs in the skin of the negro. These do not affect the situation to any great extent. The great and only real difference is the excess of pigment, which is as marked microscopically as it is to the eye in the living subject. In the blonde the pigment is in the lowest part of the lowest layer of the epidermis; in the negro it is so profuse as to actually stain the cells of this layer, and also the cells of the superimposed granular layer. But, whether we are dealing with the fair Caucasian or the black negro, the pigment is derived from the same source, and is of the same chemical character.

In passing, we might say that there are certain habits which are often irritative. Frequently, excessive cleanliness with soap and water will produce skin troubles. Needless to say, the negro is, as a rule, not troubled with these from a lack of hygiene as well as the excess of the amount of sebaceous material.

We are accustomed to regard sunburn as a part of the excessive heat of summer, though the heat has nothing to do with it. It is a common practice for Arctic explorers and hunters to protect their faces with a black paste when out on the ice floes at work, otherwise the light reflected from the glittering ice fields will produce a most intense dermatitis, though the temperature may fall below zero· We further know that a black veil will protect against sunburn, though the white veil will only protect partially. The negro is immune to all of these because of his natural pigment screen. This fact suggests a similar condition with artificial light, and we find the same thing. It takes much longer to produce a reaction on the skin of the negro with the X-ray, or any other of the lights used in the treatment of skin conditions. This freedom from irritation by light points to the relation between light as a cause, and certain diseases from which the negro seems to be free.

In this audience there will be a dozen men past middle life with keratoses appearing on the skin as hard, scaly nodules. We frequently see these partially degenerated, and, as such, recognize them as the beginning of skin cancer. So far as I know, no case of skin cancer has ever been reported of the true black. Of course, in this discussion, the mulatto and mixed type are excluded, though personally the same observation holds true. The comparatively rare condition called xeroderma pigmentosum, which is a sort of cancer dermatitis, occurring on the exposed parts of the body, is also unknown in the negro. The effect of light in producing these pre-cancerous conditions has been shown by Morris and others. It has been further proven by numerous cases of epithelioma, which wrought such havoc among Roentgenologists, many of whom have suffered mutilation and death due to cancer developed from the stimulating effect of X-ray. It is rather peculiar inasmuch as epithelioma can be successfully treated by X-ray. But here we are dealing with an intensifying exposure over a short period rather than continuous treatment over a long one.

The main purpose in the presentation of this paper is to perhaps add one point to the things known in the etiology of the eczema of infants. Eczema is a disease so protean in character that it is reasonable we should have as protean an etiology. We have those eczemas which are due primarily to external irritation, such as those occurring on the hands of the trained nurse from working in antiseptics, and the different dry eczemas and other conditions purely external in character. We recognize also those types which are absolutely toxic and arising from toxaemia, which, when removed, the disease rapidly heals. There are also cases of reflex nature, and one who would treat these diseases successfully must be willing to search patiently for its cause. There is one form of eczema, however, which is a bug-bear to the general practioner, pediatrist and dermatologist as well,—that form called the milk crust of infants. It usually appears shortly after birth, and lasts frequently until the child is fairly well grown. It presents certain different characteristics· When it occurs on the scalp there is usually a seborrheic involvement, but frequently it avoids the scalp, and the common sites are the cheeks and ears, extending from here over the rest of the body· It may bear no apparent relation to the child's health, for it is frequently seen in the most robust children, causing the most intense discomfort and worry to the child and those around him·

It frequently become abraded and exuding, and the raw, red surface may so alter the appearance of the child as to make it horrible to look upon. It has been generally accepted that these cases, although there are symptoms due to external causes, are, in the main, the result of improper assimilation of food, and different investigators have of late worked at length to prove this contention, their conclusions being that it is more of an anaphylaxis and that casein is more often the offending food. While this is undoubtedly true in part, it is so far from conclusive as a whole. We know that diet is a strong contributing, if not actual, cause in the appearance of pellagra, but many observers have noticed that the skin symptoms of pellagra are much increased if exposed to a great deal of light. In other words, the toxemia has sensitized the skin so that light will readily hurt it. To say the least it is suggestive that in an experience long enough and large, I have yet to see this type of eczema in the pure black baby. I have questioned many of my friends, nor can they recall any such case. The reason usually put forward for this is the lack of water and soap as an external irritant, but this, to my mind, does not cover the case. There is no class of babies so horribly neglected in a dietetic sense. The little negro baby is frequently given food from the parent's table when it is at the breast, and the cases of rickets and malnutrition are more common here than among the whites. It would certainly seem that if food were the main cause, it would be more common with the tar babies than with the whites. This makes me believe that light is one of the main causes of eczema of infants, though no doubt acting on a skin sensitized by food toxins.

I shall at some future time bring to you the results of experiments which I intend to carry out in an attempt to prove, or disprove, this theory. I bring it to an issue now with the idea that discussion might help me with the work, and possibly lead to a report of cases that would contradict some of the ideas herein suggested.—The Virginia Medical Semi-Monthly.

### MEDICAL PREPAREDNESS

In a broad sense every earnest medical student and every studiously observant medical practitioner is preparing for the war which most people think inevitable and imminent—that is, he is preparing for his particular part in the war; nor in the case of the surgeon at or past middle life if further preparation needful or desirable, since such ones should be used either as consultants or directors in their purely professional capacity, or should be given the care of patients discharged from base hospitals to the interior.

On the younger professional men will fall a more varied responsibility: (1) The duty of examining recruits—that is, of selecting men physically fit to stand a vigorous course of military training and to profit by it. (2) The early recognition of those who soon show that they are physically or mentally unfit. (3) The protection of the soldiers against disease; this implies a broad knowledge and a rigorous application of the rules of military sanitation· (4) The immediate care of those crippled in the service, their prompt and safe conveyance to dressing stations or hospitals; this includes the whole broad question of transportation. (5) A working knowledge of the record system obtaining in the army. (6) A practical knowledge of food; its initial value, standards of excellence, and best methods of preparation.

It is obvious that a medical education even a broad one supplemented by specialization in surgery, re-enfcred by a large patriotism and the wish to help, are not enough to fulfil the requirements, even as sketchily outlined above. The work is distinctly technical in many of its phases and requires a specific, direct, and practical preparation; in part provided for by the United States in its camps at Plattsburg and Tobyhanna, in part by its correspondence school, in part by experience gained in service with the National Guard.

It is obvious that complete efficiency can be attained by the thousands of the younger medical officers who will be called out to active service only by actual experience, and it is equally obvious that this efficiency will come earlier and be more complete in direct proportion to the painstaking efforts to prepare in so far as this is possible in times of peace, nor does this preparation consist in the purchase of a uniform and the receiving of a commission in the Reserve Corps. Every man who believes he is still young enough to play an active part in the coming war should attest the sincerity of his conviction by availing himself to the full of the opportunities offered by the Army Medical Corps.

Aside from personal preparedness there is preparedness concerning material needed. Thus, at the suggestion of the National Red Cross, numbers of base hospitals have been organized. These are usually in connection with teaching institutions and with the thought in mind that thus might be assembled a staff of more than average experience and so used to each other's ways that team work might be expected from the beginning. Such a base hospital was mobilized in Fairmount Park on the last day of the meeting of the American College of Surgeons in Philadelphia. The army furnished the tentage, which was put up with extraordinary speed by Major Harold Jones with a small corps quite untrained as a working team. The equipment was sent over from New York from the Presbyterian Hospital, their unit under Dr. George Brewer having purchased the $25,000 worth of material needful for the supply of the base hospital. The personnel was brought on by Dr. George Crile, of Cleveland, as representing the Lakeside unit. This hospital was prepared to receive patients and could have adequately cared for them. In itself it was of little service beyond a practical demonstration of the earnest purpose of those advocating preparedness, and unless it is followed by something very much better and more complete—for instance, such a base hospital as was exhibited in model by Brewer—it will have fulfilled no largely useful purpose other than that incident to the demonstration of the difficulties and delays attendant upon any mobilization.

Dr. F. F. Simpson is now engaged in a careful inventory of the medical resources in the United States, including both hospitals and medical and surgical supplies. Dr. Bloodgood and his committee are concerned in solving the question of the most efficient first-aid package. Dr. Estes and his committee are endeavoring to standarize the treatment of fractures.

The thousands of surgeons of both the Central Powers and the Allies have as yet failed to give us the answer as to the best treatment of lacerated, contused, and infected wounds aside from free drainage.

It will be well for each medical man to set clearly before himself the thing for which he wishes to be prepared. For the home service the educated man is ready; for field service he requires a complete and extensive education. If he anticipates field service the more quickly he gets the rudiments of this education in time of peace, the more surely will he attain efficiency and honors when the need comes.—The Therapeutic Gazette.

---

### MEDICAL SERVICE IN THE ARMY AND THE NAVY.

**By W. H. Newcomb, M. D.,**
New York.

Preparedness is the topic of the day, and it is fitting, nay, essential, that the army and navy shall be provided with an adequate and efficient medical service. In this brief article an attempt will be made to describe the work and life of the army and naval medical officer, and to draw some points of comparison between the opportunities afforded by an army and civil medical career.

It goes without saying that there is no space to enter into exhaustive details. It may at once be stated that the army and naval medical departments are undermanned, although the members thereof are efficient and skillful. Consequntly the object of those responsible for the introduction of reforms is undoubtedly to place the army and naval medical departments on a satisfactory basis, from the standpoint both of numbers and of efficiency. The navy in particular is lacking in medical officers. The condition of the British army medical service at the time war broke out was somewhat analogous to that of the American army at the present time. The medical corps was barely sufficient for the standing army of Great Britain, but was wholly inadequate when that army was immensely enlarged. Therefore, civil practitioners were called upon, as they have been called upon to a much less extent in this country. However, it is not the intention here to criticize the organization of either the army or navy medical service, even if such criticism were justified, but rather to endeavor to show the kind of career offered to an ambitious and energetic young medical graduate or indeed to an older man drawn from civil life. Further remarks will be confined to a consideration of army medical conditions as far as the duties and opportunities of the medical officer are concerned.

The medical department of the Army now consists of the Medical Corps, the Reserve Corps, the Dental Corps, the Hospital Corps, and the Nurse Corps. The Medical Corps consist of a surgeon general with the rank of brigadier general, twenty-two colonels, thirty-six lieutenants, with the rank, pay, and allowances of officers of corresponding grades in the cavalry branch of the service.

### General Requirements For Application.

An applicant for appointment in the Medical Corps of the Army must be between twenty-two and thirty-two years of age, a citizen of the United States, and a graduate in medicine of a reputable medical school. He will be required to submit his diploma to the board at the time of his preliminary examination.

Hospital training and practical experience in the practice of medicine, surgery, and obstetrics are essential, and an applicant will be expected to present evidence that he has had at least one year's hospital experience as an interne after graduation. Marriage makes no difference with respect to his eligibility. The examination consists of two parts, a preliminary examination and a final, or qualifying, examination.

### Preliminary Examination.

The preliminary examination consists of a physical examination, which is thorough, and a written examination on professional subjects. Candidates who are not sixty-four inches in height are rejected. Each candidate is also required to certify that he suffers from no physical infirmity or disability which may interfere with the efficient discharge of his duty. Errors of refraction, if vision is not below 20|100 in either eye, are not causes for rejection, provided that they are not accompanied by ocular disease and are entirely corrected by appropriate glasses. The standards of height and weight are fairly high, but, of course, as to these points the members of the examining board are vested with a considerable amount of discretion.

The written examination embraces the following subjects: anatomy, physiology and histology, chemistry and physics, materia medical and therapeutics; surgery, practice of medicine, obstetrics and gynecology.

The preliminary examinations are conducted under instructions from the Surgeon General by local boards of one or more medical officers and by a central board of not less than three, which is known as the Army Medical Board. Applicants who attain a general average of not less than eighty per cent in the preliminary examinations and are deemed otherwise acceptable, will be appointed to the Medical Reserve Corps with the rank of first lieutenant and ordered to the Army Medical School, Washington, D. C., for instruction as candidates for admission to the Medical Corps of the Army. A selected applicant will be required before entering the school to make an agreement to accept a commission in the Medical Corps if found qualified in the final examination and serve at least five years thereafter, unless sooner discharged. An applicant failing in one preliminary examination may be allowed another after the expiration of one year, but not a third.

The course of instruction at the Army Medical School is of eight months' duration, commencing on the first of March next succeeding the preliminary examination. It is both theoretical and practical and comprises the following subjects: duties of medical officers, medical department administration and customs of the service; military hygiene; clinical microscopy and bacteriology; military surgery; military and tropical medicine; sanitary chemistry; hospital corps drill and field work; operative surgery; ophthalmology and optometry; x ray work; equitation. Great attention is paid to the conduct of the applicant while studying at the Army Medical School, and if he fails to qualify for graduation conformably to the regulations of the school he will be relieved from active duty and his discharge from the service recommended at the close of the term of the school. A second course in the school will in no case be allowed.

### Final Examination.

The final, or qualifying, examination of graduate candidates for appointment in the Medical Corps will be held by the Army Medical Board immediately after the close of the term of the Army Medical School. It will cover the following points: the candidate's physical qualifications, his clinical skill and acumen, and his general aptitude for the service.

### Appointments.

Graduate candidates who are found physically qualified and who obtain a general average of eighty per cent. in their preliminary professional examination, their course at the Army Medical School, their clinical examination and their general aptitude will be elegible for appointment in the Medical Corps. Eligibles candidates who fail to receive appointments because of lack of vacancies at the time of qualification may receive them in the order of their standing as vacancies occur before the graduation of the next class. Thereafter they shall not be eligible for appointment in the Medical Corps, but will be preferred for selection for volunteer commissions and for active duty in the Medical Reserve Corps.

### Salaries and Perquisites.

The pay is generous. A first lieutenant receives $2,000 a year. At the end of five years he is promoted to captain and receives $2,400 a year. In two years more he gets $2,640 yearly, so that after ten years' service the pay will be $2,880 annually. The pay attached to the rank of major is $3,000 a year, which with ten per cent. added for each five years' service becomes $3,600, after ten years' service, 3,900, after fifteen years' service, and $4,000 after twenty years. The monthly pay of lieutenant colonel, colonel and brigadier general is $375, $416,-66 and $500 respectively. Officers in addition to their pay proper are furnished with generous quarters, according to rank, either in kind, or where no suitable government building is available, by commutation· The commutation for quarters, light, and fuel for a first lieutenant amounts to approximately $500 a year, and increases with each elevation in rank. This allowance, when added to the commutation for forage where the officer is on duty that does not require a horse, makes a considerable addition to the income. Officers when traveling on duty are provided with transportation, while all officers of the Medical Corps are provided with forage, stalling, and transportation for horses owned and actually kept by them, not exceeding two for all ranks below that of brigadier. Horses and horse equipments are furnished by the Government for all mounted officers below the rank of major. Groceries and other articles may be purchased from the commissary at about whole-sale cost prices. Instruments and appliances are liberally supplied for the use of medical officers in the performance of their duties. Well selected professional libraries are part of the equipment of each hospital and at each military post there is a laboratory for those interested in such work. Medical officers are encouraged to carry on any special study which appeals to them and which tends to fit them better for their work as medical officers.

Before discussing the possibilities and potentialities of army medical service as a career it will be quite apposite to say a few words with regard to other possibilities.

### Preventive Medicine In The Medical Service.

The most important part of the duties which fall within the province of the military surgeon is undoubtedly that of hygiene. It is no exaggeration to say that since 1870 surgery and preventive medicine have become new sciences, but it is especially in the development of preventive medicine that the greatest strides have been made. There are said to have been 21,000 cases of enteric fever in the American volunteer camps during the Cuban wars and all wars have had similar accompaniments. The ancients saw that "prevention is better than cure" cannot be more aptly applied than in the case of the magnificient work done by military preventive medicine. Up to the time that military hygiene had attained its present high degree of excellence, disease exacted by far a greater toll of human life than shot and shell. It is accordingly upon the preventive side of their work that the medical officers of an army mainly rely. The principals of good santitation put into practice with zeal tempered by discretion will effect more in the saving of life and prevention of disease than any other means. Thus it behooves the army surgeon in the making to learn very thoroughly all that there is to be learned concerning this subject. It is satisfactory to know that every opportunity is afforded for the young man entering the Medical Department of the Army at the present time to obtain the best instruction in this most essential branch of his duty.

### Surgery In The Medical Service.

In modern worfare every mode of treatment must be provided for, from dentistry to massage. War surgery is no longer a more or less straightforward affair dealing with the dressing of wounds.

### The Dental Corps.

The Dental Corps of an army is of the utmost consequence and the truth of this statement has been most clearly demonstrated by the manner in which plastic surgery has developed during the progress of the war in Europe. The most appalling injuries to the jaw have been repaired in an almost marvelous way. A great deal of the best work done in this direction in Paris has been due to the skill of American dentists. But in time of peace the dentist is necessary. The care of the teeth and oral hygiene generally are now an indispensable means of preserving the health. It is gratifying to learn then that the Dental Corps has been reorganized and the probationary contract system abolished. Dental surgeons are now commissioned as first lieutenants and after eight years' service , promoted to captains. It is further provided that after twenty-four years' service a number of dental surgeons not exceeding fifteen in number may be promoted to the rank of major.

### The Veterinary Corps.

Another notable advance in connection with the reorganization of the Army Medical Service is that a Veterinary Corps has been established and has become an integral part of the Medical Department. Appointments are made to the Veterinary Corps as assistant veterinarians with the rank of second lieutenants. After five years' service a second lieutenant is promoted to first lieutenant. The law provides that after fifteen years of service he attains the rank of major.

### The Army Nurse Corps.

The reports of the work of the Army Nurse Corps have been highly gratifying and an increase of the corps has been authorized for the coming year. Owing to the mobilization of the regular troops and militia many hospitals have been established on the border, and the appointment of 276 nurses was authorized to meet the emergency. Further, nurses have been and are being appointed in the regular corps as rapidly as possible to meet the need, and their numbers have been augmented by the assignment to active duty of a large number from the reserve list, the enrolled nurses of the American Red Cross constituting this reserve.

The Army Medical Service undoubtedly offers many inducements to the energetic, virile young man. There may not be the big prizes from the monetary point of view that are open to the practitioner in civil life, but, on the other hand, in the army there is an assured income, and on the whole it may be stated that the young army surgeon earns more and has a more congenial life than the struggling youth civilian practitioner.

### The Medical Reserve Corps.

The Medical Reserve Corps number 2,400 according to the latest roster, of whom 146 were on active duty and 2,254 on the inactive list.

An officer of the Medical Reserve Corps residing in the vicinity of each military post has been designated as a locum tenens, and the policy has been adopted of calling such officer into actual service for duty at his post when all the regular medical officers thereat are called upon to accompany troops into the field. It will be observed that the principle underlying the establishment of the Medical Reserve Corps was to supply, as far as was possible the manifest shortage of medical officers of the army. The Medical Reserve Corps is then a compotent part of the army. One year after the passage of the act (1916) for making further and more effectual provision for the national defense and other purposes, the Medical Reserve Corps as now constituted by law shall cease to exist. Members thereof may be commissioned in the Medical Department of the Officers' Reserve Corps, subject to the provisions of this act, or may be honorably discharged from the service.

The Secretary of War may in time of peace order first lieutenants of the medical section of the Officers' Reserve Corps, with their consent, to active duty in the service of the United States in such numbers as the public interests may require and the funds appropriated may permit, and may relieve them from such duty when their services are no longer necessary. While on such duty they shall receive the pay and allowances, including pay for periods of sickness and leaves of absence, of officers of corresponding rank and length of active service in the Regular Army.

As a matter of fact, they are practically officers of the Army under the same regulations and entitled to the same privileges as all other officers of the Officers' Reserve Corps remain in force for a period of five years unless sooner terminated at the discretion of the President. Such officers may be recommissioned, either in the same or higher grades, for successive periods of five years, subject to such examinations and qualifications as the President may prescribe and to the age limits prescribed in accordance with military regulations; provided, that officers of the Officers' Reserve Corps shall have rank therein in the various sections of said Reserve Corps according to grades and to length of service in their grades. Thus when this act goes into force the Medical Reserve Corps will be on the same footing to all intents and purposes as the Officers' Reserve Corps, of which its members form a part and which again is a part of the Army. The formation of the corps on these lines is an intelligent effort to organize the civil practitioners who are willing and anxious to serve their country, so that they may be able to serve her in the most efficient manner. It is plainly evident that without a certain amount of training the general practitioner drawn from civil life is not equal to the fulfillment of his medical military duties as the man who has been accustomed to army work. It is not enough to be a good surgeon or an excellent all round medical man in order to succeed in the army. A great deal of military medical work is peculiar to the army and cannot be learned in a day or in civil life. Therefore to be prepared for all eventualities it is essential that the patriotic civil medical man who has declared his willingness to assist his native land should be efficient in his military medical duties. At the present time the means for gaining the necessary instruction are adequate for those who are desirous of improving their military medical knowledge.

That is to say, so few members of the Medical Resreve Corps as now constituted have taken advantage of these facilities that the opportunities provided are ample. Instruction is given in army medical schools, and in camps of instruction of which little advantage has been taken. Prabably the officers of the Medical Reserve Corps have either felt that they could not spare the time or have failed to appreciate the need for expert medical military knowledge and have not realized their own responsibility.

It is true, however, that recently much more interest has been displayed in this direction, but nevertheless it was felt that a correspondence course would interfere least with their professional work. Accordingly, upon the recommendation of the Surgeon General, the Adjutant General of the Army directed on September 7, 1915, that a correspondence course for officers of the Medical Reserve Corps should be conducted by the director of the Field Service and Correspondence School for Medical Officers, Fort Levenworth, Kansas. The correspondence course commenced on October 1, 1915, and it is intended that it shall cover four annual sessions, beginning each year on October 1, and ending on April 30, with an optinal post graduate course of one session during the same part of a fifth year. A new course will be begun each year to furnish an opportunity for new officers or nonparticipants, to take up the progressive course from the beginning. According to Major M. A. W. Shockley, M. C., U. S. A., director of the Field Service and Correspondence School for Medical Officers, Fort Levenworth, Kansas, writing in **The Military Surgeon,** January, 1916, arrangements will be made whenever possible for a written examination on the subjects covered by the year's work. The subject of this correspondence course include most of those which come within the sphere of a medical army officer's duties and "certificates of proficiency" will be given to the officers who have acquitted themselves well in the course and examination, that is, so far as the subjects they have completed are concerned.

Correspondence papers will be forwarded to officers at such times as will permit them to have at least three weeks for consideration and reply to the questions or problems before the date of their return. As Major Shockley remarks at the conclusion of his paper, "the manner in which the work has been taken up is most satisfactory, and it is believed that the officers participating will get an insight into their duties, as medical officers, that will be of material advantage to them, and to the United States, when ordered into active duty." From a persual of this somewhat cursory account of the Army Medical Reserve Corps, it may be gathered that there are a goodly number of patriotic practitioners of medicine and surgery in the United States who belong to it and that schemes now afoot for their organization and instruction will doubtless secure the required affieiency.

In **The Military Surgeon** for February, 1917, is printed the Wellcome First Prize Essay, 1916, by Captain Mahlon Ashford, Medical Corps, U. S. A., dealing with the organization, training, and utilization of the medical officers of the Army and Navy Medical Reserve Corps. In this remarkably able disquisition of a question which he assuredly has at his fingers' end, he points out in his opinion the ideal mode of dealing with the matter.

For instance, he lays particular stress on the point that the proper place to commence the instruction of medical men for military emergencies is the medical school. Here, he goes on to explain, we can reach every potential doctor, not an occasional one as under the existing system. The students are chiefly young men whose sympathies may be enlisted more readily and whose ideals are untarnished by rubbing too much with the world. They are in a receptive state and are rapacious for knowledge and therefore easily acquire it.

The military service in the Regular Army or in the Navy offers an attractive career for the young doctor who likes change and adventure.

The older medical practitioner—there is no age limit—who wishes to serve his country in case of war and the younger man who does not care to devote his whole life to the Army, should join the Medical Reserve Corps, making application to the Surgeon General of the Army or of the Navy at Washington. Membership in this corps will enable the civilian to fit himself for active duty, and he cannot be called to active duty without his consent, except in case of war, so that in case of war he will be able to render the greatest measure of service to his country. The Medical Reserve Corps now includes many of the most distinguished surgeons and practitioners in the United States.

157 West 105th Street.

—**The New York Medical Journal.**

---

### BLOOD-PRESSURE AND THE WAR NEWS

No doubt you read the war news as assiduously as your neighbor, and no doubt you have been lured into quite a number of discussions by views expressed in your presence that have irritated you because they were not in consonance with your own. While the discussions were on, you may have been much calmer than your opponent, and may have remarked afterwards how easily moved to shouting he is. But granting that you did not display his tempestuous outbursts, that even when goaded on by his asinine remarks you held yourself in check, the fact remains that despite your outward calm, for which you deserve a great deal of credit, there must have been a number of upheavals within you of which you were not cognizant, or rather which you made light of. Upheavals would be of small moments were they to occur at long intervals, but the Great War has been on over two years, and every day means for the intelligent and ''reading'' man a new set of ideas and a number of possibilities of meeting some one who will attempt to combat them. The fires within you have at times been low and again they have been burning briskly; the cauldron may have been cool, even cold, and again red-hot; your daily avocations may have gone on just the same. But have you ever given a thought to your blood-pressure? Of course, you have not, though it can be said with considerable assurance that in the two and a-half years the war has been on, you have sent a large number of patients to doctors who specialize in blood-pressure, or have taken it yourself. Such is human nature.

But let us admit that you are a stoic in all discussions; that no matter what is said about the Allies or the Germans the Mona Lisa smile never deserts your lips; that you are so decidedly neutral that you have become an insufferable bore to all your friends. Are you the same sort of stoic directly you read the morning paper? Let us suppose that you are pro-Ally, and that you have read in the evening paper that the French and English smashed the German line and took 20 yards of good French earth and some two hundred German prisoners. You went to bed in a joyful mood. The following morning you are completely disillusioned, for the morning paper states absolutely nothing about the French and English having been victorious, but offers positive assurance to its readers that the English and French lines were smashed and that the Germans took some three hundred prisoners. Do you mean to say this contradictory news leaves you unaffected, what with your sympathies for the French and English? Are you adamantine against such onslaughts on the part of the newspapers? Is your blood-pressure going to remain normal? Will the Mona Lisa smile remain fastened to your lips? Surely not; you are human, and while we grant you repose in all discussions, the reading of such news as we have set forth will perforce make your blood move faster, will put you in a more or less excitable frame of mind, will make you more active than is your wont. And before you can get back to your compulsory normal state of outward calm and the cool cauldron within and the Mona Lisa smile, the early editions of the afternoon papers have appeared on the streets, and again you gulp down news that upsets what you read in the morning paper, news that is contradicted in all the later editions of the afternoon papers. And yet you have not had your blood-pressure taken!

Medical men who write for the medical press and also for the lay press have certainly been remiss in their duties not to have given thought to the subject of the decided rise in the blood-pressure of the American people at the present time. Why they should have been remiss on so interesting a subject, is not within the ken of the writer of these lines; but it shows that with all their mental acuteness and their desire to spring something new on the public, they have failed to consider this subject in their ephemeral literary offerings. That it is a subject fraught with seriousness cannot be gainsaid; and to prove this assertion all that is necessary now is for a writer who is enamored of statistics to go to work at once on some dozen or two of his patients whom he knows to be rabid adherents of the Allies or the Germans, and directly after their reading of the morning paper institute the taking of their blood-pressure. This again should be followed by a close examination of the readings of the pressure during the whole day; and after a close and scientific observation covering some weeks, noting, of course, in his published report just what sort of war news caused an increase or decrease, would not the outcome be of benefit to the public? In truth, altogether too much thought has been given in these war times to our stomachs; the price of food is always with us, the price of clothes dangles in front of our eyes continually, especially when the nap on the collars of our overcoats and suit coats cries out the warning that new clothes must be bought or our friends will remark our shabbiness; the whole cost of living is a matter that has made even the laziest amonst us, think. But why this complete concentration on the stomach and on the problem of being well dressed on the least possible outlay? Why this gross materialism at a time when the slaughter-house of the European battle-field illustrates the refinements and complete spirituality of modern civilization? Surely the taking of blood-pressure under the present circumstances would fit in nicely with this onsweep of spirituality—Medicine and Surgery.

### NEW STUDIES OF THE ALCOHOLIC PROBLEM.

By T. D. Crothers, M. D.

Hartford, Conn.

The alcoholic and inebriate presents a most confusing mass of symptoms which follow no special order that can be defined or traced with uniform regularity. If alcohol was the only cause, there would be uniform effects. If alcohol was the only cause there would be a resemblance of symptoms and a continuous line of degeneration with but slight variations. This does not follow. One man drinks to great excess for brief periods, then abstains and begins again. After a time the free intervals grow shorter and other diseases come on. Another drinks continuously, sometimes in excess usually in so called "moderation" through a long period of years. Other diseases may appear, but they are of a low chronic grade which simply intensifies the ordinary course of events. A man, after a lifetime of sobriety, drinks to great excess and dies soon after from it. Another drinks in early life, abstains until nearly the close of life, then shows an insane thirst which is fatal. Under all conditions and all circumstances, persons drink spirits and abstain. Quack cures of every description boast of patients restored by grotesque means and methods. Injuries and diseases of almost every grade and description are followed by the excessive use of spirits in persons previously temperate. In one family there is an appearant inheritance of dringing habits of the parents. In another there is complete immunity. Children of excessive drinking parents, are strict abstainers. In still other families a sudden outbreak of the drink craze occurs two or three generations removed from drinking ancestors. Evidently there are causes back of these, confusing conditions and these wide variations in effects, are due to these causes. It is not alcohol, but something back of it. Past explanations do not explain and do not account for these phenomena. There are puzzles, paradoxes, strange anomalous symptoms that evidently are influened by some unknown forces. This is the new field for study and research work. The facts are innumberable and can be found in almost every home in the country and are within the observation of every physician. No one has ever attempted to determine what they mean. Nn one has ever grouped them with a view of tracing back thre causes. Up to the present all efforts, both sociological and scientific, with the one exception—the study of alcohol, have all been concerned with the effects and treated them as active causes. The laboratories have gone back of current opinions and found out why alcohol is attractive and how it affects cell and tissue. This had opened a new world, changing all our present conceptions. The research foundation at Hartford is organized for a new work, back of all present theories and conceptions and inquiry, what the causes are that develop the drink and drug craze. It assumes that there are causes and conditions, which can be found, that will account for these physiological and psychological riddles and puzzles which confront both the layman and physician. Evidently there is a new field here, rich in facts, but is postive and clearly a realm of laws which are traceable. The magnitude of the alcoholic evil and its intense personal interest suggests that there must be something beyond theory to explain its presence. Just as yellow fever was traceable to germs, carried by mosquitoes, so alcoholism and inebriety will be found to follow some unknown sources and to have a life history of beginning development and decline. The present theories all start from alcohol and its effects. The assumption is, that when this cause is removed the evils will disappear. This is not verifiable and fails to stand the test of experience. We want accurate data of the conditions farther back of the kind of soil and surroundings that favor the growth of this evil. We want to know something of the germ causes that in such surroundings will grow and develop alcoholism and inebriety. This is the last great move to lift the subject into the realm of science and inquire with careful scrutiny, what these forces are that produce the evils from this source. Already this research foundation has opened the door and now it goes before the profession inviting cooperation and assistance to make new discoveries and point out methods of correction and prevention that were unknown before. All that is needed is vigilant work and expert study above theories and opinion. Such a study will open a new field for consultation, examination and advice to a large army of active men who are more or less alarmed that they are using spirits at intervals or continuously with the possibility of fatal results in the future. To such men this foundation offers a hope of knowing how they can extricate themselves and with the aid of the family physician, fully recover. It is literally, the first aid to the wounded. Stop the disease at the beginning, but this can only come from a knowledge of the causes and what the beginning are. This is a field and this is a great new work, that is most urgently called for at present.—**The Medical Fortnightly and Laboratory News.**

